# Understanding the Role and Impact of Poly (Ethylene Glycol) (PEG) on Nanoparticle Formulation: Implications for COVID-19 Vaccines

**DOI:** 10.3389/fbioe.2022.882363

**Published:** 2022-06-07

**Authors:** Esperanza Padín-González, Pearl Lancaster, Massimo Bottini, Paolo Gasco, Lang Tran, Bengt Fadeel, Terence Wilkins, Marco P. Monopoli

**Affiliations:** ^1^ Department of Chemistry, Royal College of Surgeons in Ireland (RCSI), Dublin, Ireland; ^2^ Department of Experimental Medicine, University of Rome Tor Vergata, Rome, Italy; ^3^ Nanovector Srl, Turin, Italy; ^4^ Institute of Occupational Medicine, Edinburgh, United Kingdom; ^5^ Institute of Environmental Medicine, Karolinska Institutet, Stockholm, Sweden; ^6^ School of Chemical and Process Engineering, University of Leeds, Leeds, United Kingdom

**Keywords:** PEG, mRNA vaccines, complement, safe-by-design, nanoparticles

## Abstract

Poly (ethylene glycol) (PEG) is a widely used polymer in a variety of consumer products and in medicine. PEGylation refers to the conjugation of PEG to drugs or nanoparticles to increase circulation time and reduce unwanted host responses. PEG is viewed as being well-tolerated, but previous studies have identified anti-PEG antibodies and so-called pseudoallergic reactions in certain individuals. The increased use of nanoparticles as contrast agents or in drug delivery, along with the introduction of mRNA vaccines encapsulated in PEGylated lipid nanoparticles has brought this issue to the fore. Thus, while these vaccines have proven to be remarkably effective, rare cases of anaphylaxis have been reported, and this has been tentatively ascribed to the PEGylated carriers, which may trigger complement activation in susceptible individuals. Here, we provide a general overview of the use of PEGylated nanoparticles for pharmaceutical applications, and we discuss the activation of the complement cascade that might be caused by PEGylated nanomedicines for a better understanding of these immunological adverse reactions.

## Introduction

Poly (ethylene glycol) (PEG) is a non-ionic polyether compound and has been widely used in common applications in cosmetics, food products, and pharmaceuticals for its excellent properties as a solvent, plasticizer, surfactant, base, and lubricant (D’souza and Shegokar, 2016). In pharmaceutical applications, PEG has several advantages as it increases the circulation time of the drugs and decreases their immunogenicity. PEG increases the molecular weight (MW) of pharmaceutical products such as proteins or peptides, decreasing the kidney clearance, and protects them from proteolytic degradation, thus altering their pharmacokinetic profile. Moreover, as a consequence of the water cloud surrounding the polymer, PEG improves the solubility of drugs. Finally, the use of PEG for the surface modification of nanoparticles (NPs) has been reported to decrease protein adsorption, thereby reducing recognition by the mononuclear phagocytic system (MPS) (Fishburn, 2008; Zhang et al., 2014).

**FIGURE 1:**
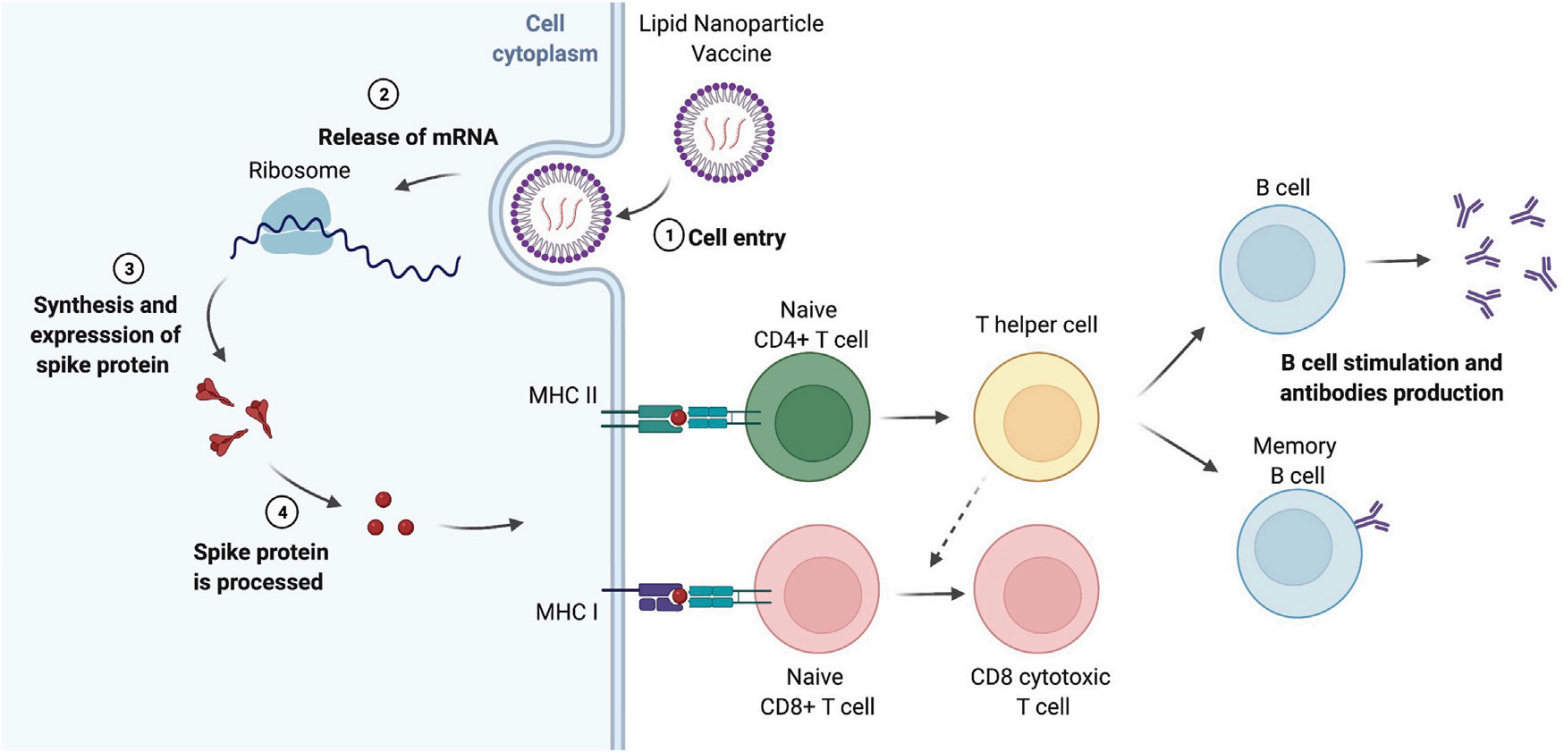
Lipid nanoparticle-based vaccines. After intramuscular injection, lipid nanoparticle (LNP) vaccines are recruited by tissue-resident dendritic cells (DCs) or resident-somatic cells (muscle cells), which translate the mRNA into proteins that are processed leading to the generation of peptide epitopes derived from the spike protein of the virus (Rijkers et al., 2021). The antigens are presented on MHC I (in the case of resident-somatic cells) and MHC II (for antigen-presenting cells including DCs) to naïve CD8^+^ T cells and naïve CD4^+^ T cells, respectively, which elicit the immune response against SARS-CoV-2. T helper cells differentiated from naïve CD4^+^ T cells stimulate the proliferation and differentiation of B cells in memory B cells and plasma cells, the latter secreting antibodies against the spike protein. On the other hand, naïve CD8^+^ T cells, with the assistance of T helper cells, produce CD8 cytotoxic T cells, responsible for the killing of the infected cells. Memory T cells (not shown) are also generated, and they are also important for the long-term protection against infection. It is worth remarking some LNPs can drain to the closest lymph nodes and interact, directly, with the resident immune cells and activate the T and B cells. Moreover, muscle cells in the injection site can also internalize the LNPs, process the mRNA and express the specific antigens on MHC class I, participating in the CD8^+^ T cell activation (Hou et al., 2021). The figure was prepared using BioRender.

PEG is the most commonly used “stealth” polymeric agent and is regarded as a safe and non-toxic polymer, gaining the *generally recognized as safe* (GRAS) status from the Food and Drug Administration (FDA) and the European Medical Agency (EMA), and it is widely used to conjugate drugs. The first example using PEG was reported in 1977, where the authors covalently attached PEG to bovine serum albumin and to liver catalase proteins, reporting changes on its immunogenicity and retaining its activity, respectively (Abuchowski et al., 1977a; Abuchowski et al., 1977b). In 1990, the FDA approved the first PEGylated protein product, Adagen, used for severe combined immunodeficiency disease and, in 1995, the first PEGylated lipid NPs encapsulating doxorubicin was released as Doxil (Supplementary Table S1). As a result of the COVID-19 pandemic (Chan et al., 2020), the scientific community has worked intensively to find an effective and practical solution to combat this infection including an effective vaccine that could be administrated widely and with low side effects. It is notable that two out of four vaccines approved in Europe are based on PEGylated NPs: the Pfizer/BioNTech (BNT162b2) and Moderna (mRNA-1273) COVID-19 formulations for delivery of mRNA vaccines. The EMA thus authorized the marketing of these vaccines in December 2020 and January 2021, respectively (Cavaleri et al., 2021), and in December 2020, the FDA approved the emergency use authorization for both vaccines (FDA, 2020a; FDA, 2020b). Only in late 2021, the EMA approved a protein-based vaccine (Novavax). These lipid nanoparticle (LNP) formulations, with a size less than 100 nm (Schoenmaker et al., 2021), encapsulate and protect the mRNA which codifies the spike protein of the SARS-CoV-2 virus. The carrier brings the mRNA into the cells, and the mRNA released in the host cell synthesizes the spike protein, which, in turn, is presented to immune cells to elicit a response against SARS-CoV-2 (Teijaro and Farber, 2021; Hou et al., 2021) (Figure 1). The vaccines are comprised of, amongst other entities, PEGylated lipids (Buschmann et al., 2021; Dolgin, 2021; EMA, 2021; FDA, 2021; Hatziantoniou et al., 2021). While these vaccines have been widely referred to as “new generation” vaccines, for those working in the field of nanomedicine, similar formulations are not novel. In fact, liposomes were first described by Bangham et al., in 1965 (Bangham et al., 1965), and were later used for the delivery of enzymes or drugs (Gregoriadis and Ryman, 1971) and, almost 30 years ago, in 1993, their use as vaccines was reported in a preclinical study. This mRNA vaccine was able to induce virus-specific cytotoxic T cell responses against the influenza virus, and became a quite attractive formulation to tackle the uncontrolled viral infection globally (Martinon et al., 1993). For an overview of the currently available COVID-19 vaccines, see (Kisby et al., 2021).

PEG provides several advantages with respect to the pharmacological profile of drugs/NPs. However, some drawbacks have been also reported. Although widely considered non-immunogenic and biocompatible (Richter and Åkerblom, 1983; Richter and Åkerblom, 1984), several studies have suggested the presence of pre-existing anti-PEG antibodies in healthy individuals after repetitive exposure (Armstrong et al., 2007; Garay et al., 2012; Hsieh et al., 2018; Mohamed et al., 2019). For example, Yang and Lai reported significant concentrations of anti-PEG antibodies in a substantial 42% of patients who had not previously undergone any treatment involving PEGylated therapies (Yang and Lai, 2015). Nevertheless, after receipt of the first doses of the Pfizer/BioNTech and Moderna COVID-19 vaccines in the United States, the Advisory Committee on Immunization Practices (ACIP) detected 21 and 10 cases of an anaphylactic reaction (11.1 and 2.5 per million doses administered), respectively, but with the highest incidence in patients that had already an established allergy to drugs (CDC, 2020; CDC, 2021). The anaphylactic symptoms were speculated to be due to CARPA (complement activation-related pseudoallergy), elicited by the application of PEGylated LNPs (CDC, 2020; CDC, 2021; Greenhawt et al., 2021; Iguchi et al., 2021). CARPA has been described as a non-IgE-mediated (pseudo)allergy related to complement activation, which occurs upon first contact, without prior sensitization (Szebeni, 2014). CARPA was previously described in relation to hypersensitivity reactions observed after the administration of Doxil (Chanan-Khan et al., 2003; Szebeni, 2005; Szebeni et al., 2011). The serious adverse events due to the administration of mRNA vaccines are rare and it still remains to be proven whether PEG could be main the cause of CARPA (Bigini et al., 2021). Here, we provide a general overview of the use of PEGylated NPs for pharmaceutical applications, with the main focus on the role of PEGylation for the improvement of the biological performance of the NPs, and we describe complement activation caused by PEGylated nanomedicines for a better comprehension of immunological adverse reactions, *e.g.*, CARPA. We also touch on other factors which may drive the adverse effects.

## Chemistry of Poly (Ethylene Glycol)

**FIGURE 2:**
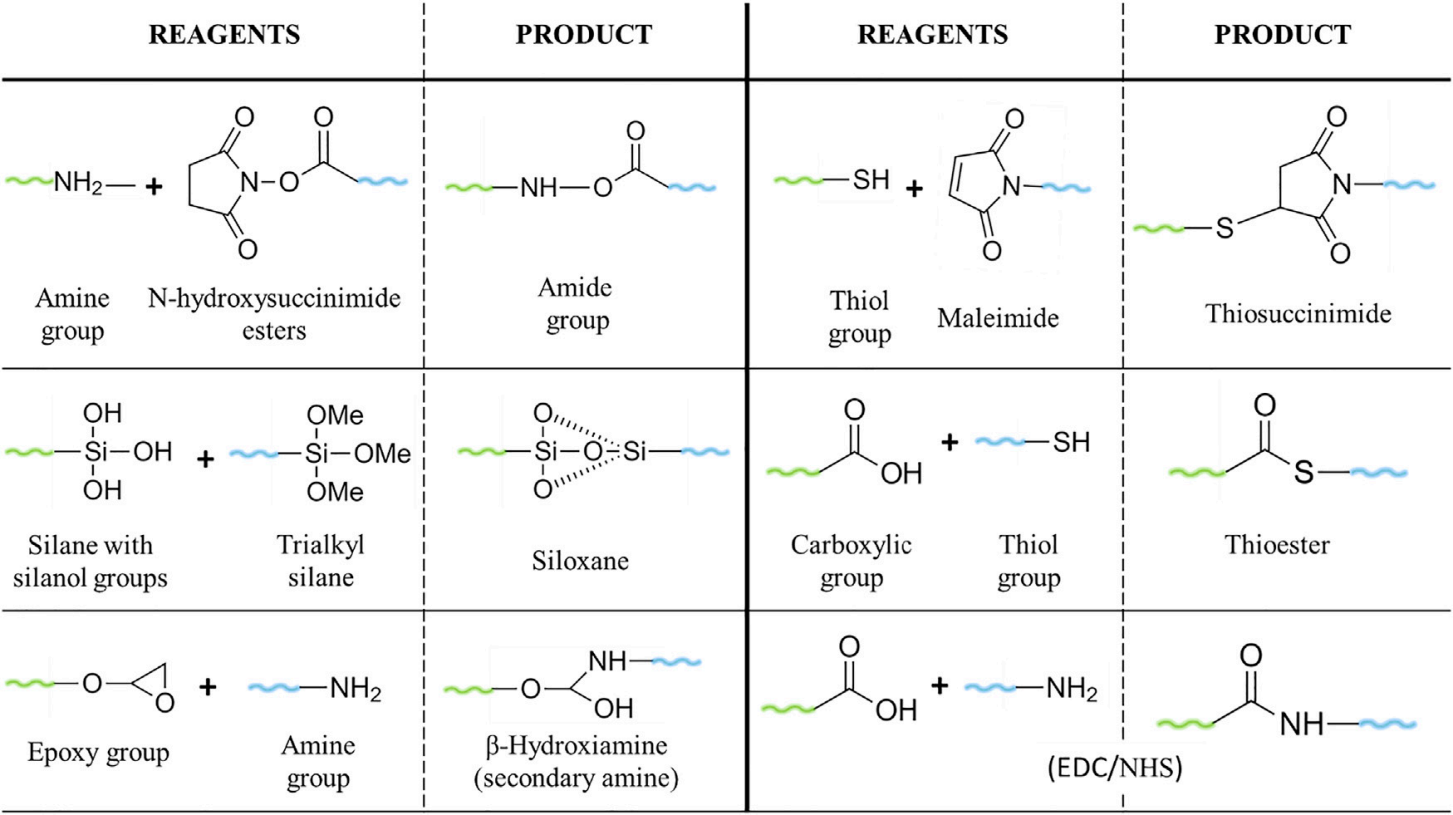
Common reactions for PEGylation of drugs/NPs.

Poly (ethylene glycols) (PEG) (HO(CH_2_CH_2_O)_n_H) are hydrophilic and non-ionic polymers synthesised *via* the polymerization of ethylene oxide under alkaline catalysis. PEG exists in a broad range of different chain lengths and MW, with a linear or branched structure (D’souza and Shegokar, 2016). The term “PEG” is often in combination with a numerical value, which refers to the average number of ethylene oxide units (n) or the average MW (g/mol) of a given PEG. The latter term is commonly used in the pharmaceutical industry. PEGylation is the term used for the conjugation of PEG to molecules, such as drugs or NPs, to enhance their biocompatibility and biodistribution (Estephan et al., 2011; Fam et al., 2020). Commercial PEGs are available in a MW range from 200–35,000 g/mol, with a narrow polydispersity (measure of the heterogeneity of sizes of PEG molecules). They are easy to produce and can be modified with different chemistry functionalities (D’souza and Shegokar, 2016) making them versatile for a wide range of bioconjugation options. PEG ranging from 20 to 50 kDa has been used for the conjugation of small molecules, such as siRNA. On the other hand, PEGs with lower MW (1–5 kDa) are preferable for the conjugation of antibodies or NPs (Knop et al., 2010). PEGylation strategies have progressed since non-specific conjugations, known as “first generation” PEGylation, which resulted in the modification of different amino groups of lysines, mixtures of isomers, impurities, or unstable and irreversible bonds; until the most recent conjugations, the “second generation” PEGylation, mainly characterized by the binding of PEG to an N-terminal or thiol in a selective and reversible way (Gupta et al., 2019; Harris and Chess, 2003; Turecek et al., 2016). Some of the most used PEGs for drug/NP modification are the N-hydroxysuccinimide esters PEG, which react with amine groups to form stable amides and the use of PEG-maleimide for the PEGylation of cysteine residues (Gupta et al., 2019). Heterobifunctional PEGs, with two functionalities in the terminal groups, allow the conjugation of ligand-macromolecules to surfaces, forming a bridge between the target ligand and the NPs (Lo et al., 2020; Huang et al., 2020). For instance, carboxylic-molecules can be attached to gold NPs using thiol-PEG-amine (Zamora-Justo et al., 2019), whereas silane-PEG-carboxylic, used for the PEGylation of silica nanoparticles, allows for the conjugation with amine groups (von Baeckmann et al., 2021) (Figure 2). Although the synthesis of PEG has improved over the years, the products of polymerization still show a complex mixture of different length oligomers, which could lead to variability in the results and/or misunderstanding in the relation between the PEG structure and the activity, bringing problems for their applications. Thus, significant efforts were made to improve the synthesis of monodisperse PEGs, although some of these approaches still present challenges regarding the low yield, complicated purification, applicability only to some MW, or expensive reagents, as well as tedious chemistry (Niculescu-Duvaz et al., 2008; Knop et al., 2010; Zhang et al., 2015; Kinbara, 2018).

## PEGylation for Biomedical Applications

One of the greatest advantages of PEGylation for biomedical applications is the high solubility in aqueous medium provided by the PEG chains (D’souza and Shegokar, 2016; Knop et al., 2010; Turecek et al., 2016). It has been reported that an ethylene oxide unit can bind two to three water molecules (Branca et al., 2002; Oelmeier et al., 2012; Kolate et al., 2014; Turecek et al., 2016). This solubility creates a “conformational cloud”, generated by the flexible polymer chains, which hides the charges and provides a steric hindrance, preventing the interaction with other PEGylated NPs, reducing aggregation and providing a high chemical-physical stability (Knop et al., 2010; Gupta et al., 2019). At the same time, the conformational freedom of the PEG chains makes the interaction with other matter thermodynamically unfavourable, decreasing the interactions of the PEGylated products with other biomolecules (Pino et al., 2014; Yang and Lai, 2015; Suk et al., 2016; Fam et al., 2020). In this regard, it has been well established that NPs in contact with the biological environment adsorb biomolecules, such as lipids or proteins, forming the so-called bio-corona. This biomolecular coating dictates the biological “identity” of the NPs, affecting their activity, biodistribution and clearance (Cedervall et al., 2007; Nel et al., 2009; Monopoli et al., 2011; Monopoli et al., 2012; Clemente et al., 2022). When these proteins are opsonins, the NPs are recognized by the MPS, and they are eliminated. However, proteins in the corona may also act as dysopsonins (Schöttler et al., 2016). The MPS is a network of cells descending from monocytes, including tissue-resident macrophages in the liver and spleen, that engulf foreign substances (particles and pathogens). The role of the MPS (also referred as the reticuloendothelial system or RES) for the clearance of NPs with a bio-corona of proteins has been discussed extensively in previous reviews (Gustafson et al., 2015; Moghimi et al., 2019).

**FIGURE 3:**
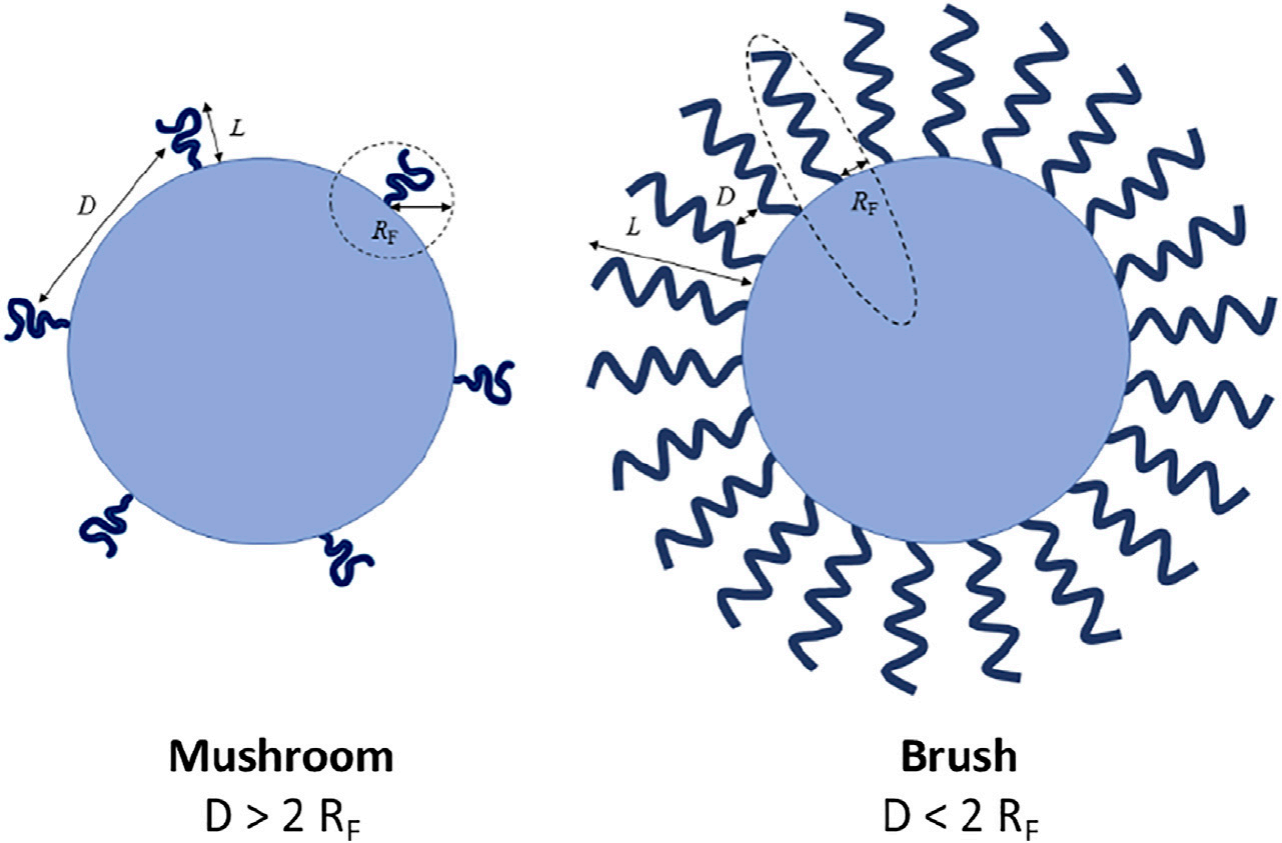
Brush and mushroom conformation of PEG on a nanoparticle surface. D: distance between two grafting sites; L: thickness of the grafted PEG; R_F_: Flory radius. Figure from (Fam et al., 2020).

The MW and surface density of PEG chains coating the NPs’ surface play an important role in the biologic performance of PEGylated NPs by regulating their biochemical (*i.e.*, protein adsorption) and physical (*i.e.*, aggregation level) properties. Increasing both PEG MW and surface density has been described to improve the NP biologic performance. Higher PEG MW provides more hydrophilicity and flexibility and a higher effect of a steric hindrance than short PEG chains, resulting in a thicker protective layer and avoiding the interactions with biomolecules (Lee and Larson, 2016). Nevertheless, there exists a limit for the MW above which the effect of PEG is negligible and, therefore, the protein adsorption cannot be completely eliminated (Gref et al., 2000). PEG density and conformation also play an important role. It has been proposed that grafted PEG can assume two different conformations, “mushroom” and “brush”, related to the PEG surface density. When the surface density is low, PEG adopts a “mushroom” configuration, where the chains are separated among them and located close to the surface. However, for higher values of density, PEG chains are closer and acquire a straighter (brush-like) conformation thus forming a thicker hydrophilic barrier that might lead to a lower protein adsorption (Walkey et al., 2012). PEG conformation can be described by Flory radius (R_F_), which defines the minimum distance required between grafted polymer chains to assume a mushroom conformation: R_F_ = *α*×n^ν^, where n is the number of monomers in the polymer chain, *α* is the length of the polymer monomer, and *ν* is Flory exponent, which is related to the solvent. Different topologies of polymer configuration have been described depending on the relationship between the grafting density and the Flory radius (Figure 3). The influence of PEG properties, including the length, packing density and the conformation assumed after binding to the NP surface has been widely studied. For instance, it was found that the density of PEG chains on the surface of single-walled carbon nanotubes mediates the adsorption of plasma proteins on the nanotubes which, in turn, affects their pharmacokinetic profile (Sacchetti et al., 2013). A change in PEG conformation from mushroom to brush transition for ovalbumin NPs affected the competitive adsorption of plasma proteins on the NPs’ surface, leading to a higher adsorption of clusterin (apolipoprotein J) and a lower adsorption of serum albumin. Thus, the brush conformation led to a shorter phagocytic uptake (Li et al., 2021a). In a similar study using PEGylated gold NPs and PEG of different MW, the authors observed that the protein corona composition for the different NPs was critical to enhance tumour targeting (Cui et al., 2021). Finally, it is worth noting that PEGylation should be carefully optimized if targeting agents are added on the NPs surface to improve the accumulation in a specific tissue and/or cell sub-population. PEG chains may cover the targeting agents and hamper their interaction with tissue/cell receptors. This problem has been tackled by coating the NPs’ surface with PEG chains of different MWs: the shorter ones were used to improve the stability of the NPs in aqueous solutions and decrease the adsorption of biomacromolecules, while the longer ones were used to bind targeting agents at their terminal end, thus allowing them to protrude from the NPs’ surface thereby avoiding the masking by other biomolecules (Cruz et al., 2011).

## Preparation of PEGylated Lipid Nanoparticles

**FIGURE 4:**
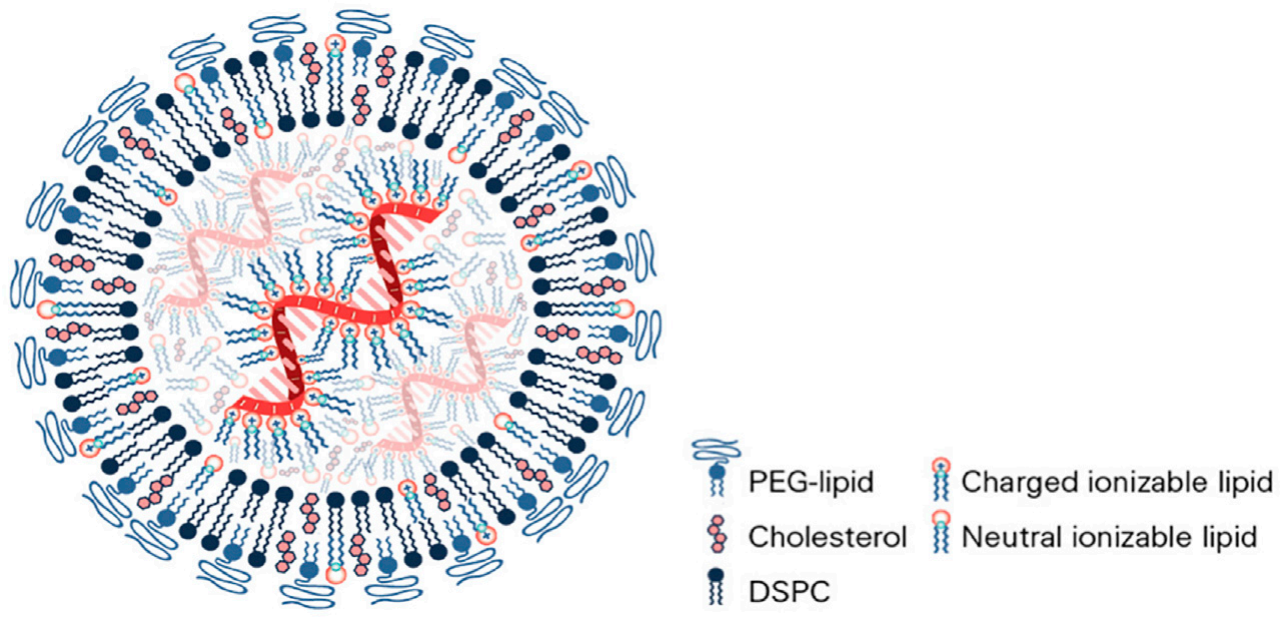
Brush and mushroom conformation of PEG on a nanoparticle surface. D: distance between two grafting sites; L: thickness of the grafted PEG; R_F_: Flory radius. Figure from ref (Buschmann et al., 2021).

PEGylated lipid NPs for drug or vaccine delivery *in vivo* are created by a simple self-assembly mechanism which normally consists of the covalent conjugation of a lipid and the chosen PEG molecule (Nosova et al., 2019). This molecule together with three other lipids (one of which is usually cholesterol) plus the active RNA ingredient will self-assemble into a lipid bilayer nanocapsule (liposome) entrapping the short interfering or messenger RNA therapeutic ingredient inside (Figure 4). The lipid bilayer is held together by hydrogen bonds between the ionic ends of the lipids. The inner and outer surfaces of the lipids are non-polar. Within this arrangement, the PEG functionalized lipid is on the outside of the lipid layer and has its polar end in the centre of the lipid bilayer. A successful and recently developed liposome production technique is microfluidic hydrodynamic focusing, in which a stream of lipid in alcohol solution is forced to flow in the central channel of a device, intersected, and sheathed by coaxial stream(s) of an aqueous phase. Reciprocal diffusion of alcohol and water across the focused alcohol/water interface causes the lipid to precipitate and self-assemble into liposomes (Tenchov et al., 2021). Controlled particle size 70 nm liposomes are achieved by pressurized reverse osmosis filtration through ceramic membranes by batch or continuous processes. The ingredients and formulations for both the Pfizer/BioNTech (BNT162b2) and Moderna (mRNA-1273) vaccines registered with EMA and the FDA are presented in Table 1 (Buschmann et al., 2021; Dolgin, 2021; EMA, 2021; FDA, 2021; Hatziantoniou et al., 2021). There are some differences in lipids and buffers used which may determine the differences observed for the stability and storage of the vaccines. However, both use PEG 2000 as the external “stealth” moiety on the surface of the liposome. Moreover, the observed albeit low incidence of pseudoanaphylaxis of the two vaccines is very similar.

**TABLE 1:** Lipid components in mRNA vaccines manufactured by Pfizer/BioNTech and Moderna.

	Pfizer/BioNTech (BNT162b2)	Moderna (mRNA-1273)	
Ionizable lipid	Acuitas ALC-0315 (4-hydroxybutyl) azanediyl) bis (hexane-6,1-diyl) bis (2- hexyldecanoate)	SM-102* (heptadecan-9-yl 8-((2-hydroxyethyl) (6-oxo-6-(undecyloxy) hexyl) amino) octanoate)	Protonated at acidic pH, allows the conjugation with the mRNA during the LNP synthesis. In the endo-lisosomal compartment, improve the endosomal release
PEG-lipid	ALC-0159: 2- [(polyethylene glycol)- 2000]-N,N-ditetradecylacetamide	Polyethylene glycol dimyristoyl glycerol [PEG 2000—DMG]	**-**
Phospholipid	1,2-distearoyl-snglycero-3 phosphocholine (DSPC)	1,2-distearoyl-snglycero-3 phosphocholine (DSPC)	Stabilizer lipid, helps to form a stable bilayer under the PEG surface
Cholesterol	**-**	**-**	Promote the fusion with the plasma membrane, filling gaps in the LNP

*Moderna proprietary.

It is notable that other constituents of the mRNA vaccines may also trigger allergic reactions. Unlike the Pfizer/BioNTech vaccine, the Moderna vaccine contains Tris, or tris(hydroxymethyl)aminomethane (tromethamine/trometamol), a compound that has been reported to cause contact dermatitis and rare cases of anaphylaxis in relation to gadolinium-based contrast agents (Klimek et al., 2021). It is thus possible that other excipients might cause hypersensitivity reactions.

## The Other Side of the Coin: Adverse Effects of PEG

### Metabolism of PEG: Potential Implications for Adverse Effects

As for any pharmacokinetics studies of conventional drugs, the fate and behavior of PEG itself should also be addressed. Parenterally administered PEG is excreted by the kidney at a rate dependent on the MW (Yamaoka et al., 1994). Moreover, PEG is slowly oxidized by alcohol dehydrogenase (ADH) (Herold et al., 1989) and by other oxidases including cytochrome P-450 (Beranová et al., 1990). The latter studies were conducted using microsomal fractions of mouse and rat livers, but the authors concluded that “the participation of cytochrome P-450 in the biodegradation of polyethylene implanted in a human body can most probably be assumed” (Beranová et al., 1990). It has also been suggested, on the basis of animal studies, that PEG metabolites produced by ADH may cause renal damage in a manner similar to the renal failure associated with ethylene glycol poisoning (Herold et al., 1982). These studies, published 30 years ago, seem to have been largely ignored in the modern era yet they could have implications for the adverse effects of PEG. Other interesting facets of PEG have emerged suggesting that PEG is not, as is commonly assumed, biologically inert. Hence, it was shown that an oligoethylene glycol substituent can enhance the potency of a ligand for a transmembrane G-protein-coupled receptor (GPCR) (Jiarpinitnun and Kiessling, 2010). The authors tethered a series of ethylene glycol oligomers to the C-terminus of an N-formylated peptide, a known ligand of the chemotaxis receptor, formyl peptide receptor (FPR). Unexpectedly, these substituents had a dramatic effect on chemotaxis, and this effect was dependent upon the length of the oligoethylene glycol substituent. It is notable that while PEG displays a long-range protein repellent effect, at short range, on the other hand, the interaction between PEG and protein can become attractive (Sheth and Leckband, 1997). Thus, PEG could potentially affect the binding affinity of ligands to their receptors. Furthermore, Luo et al. (Luo et al., 2017) found that PEGylated graphene oxide (GO) nanosheets provoked cytokine secretion in macrophages by enhancing integrin *β*_8_-related signalling. Indeed, computational modelling suggested that PEGylation, which is thought to “passivate” nanomaterials (Shi et al., 2022), may transform GO into a biologically relevant signalling entity (Zhang et al., 2021).

### Immunogenicity and Haematological Impact of PEG

**FIGURE 5:**
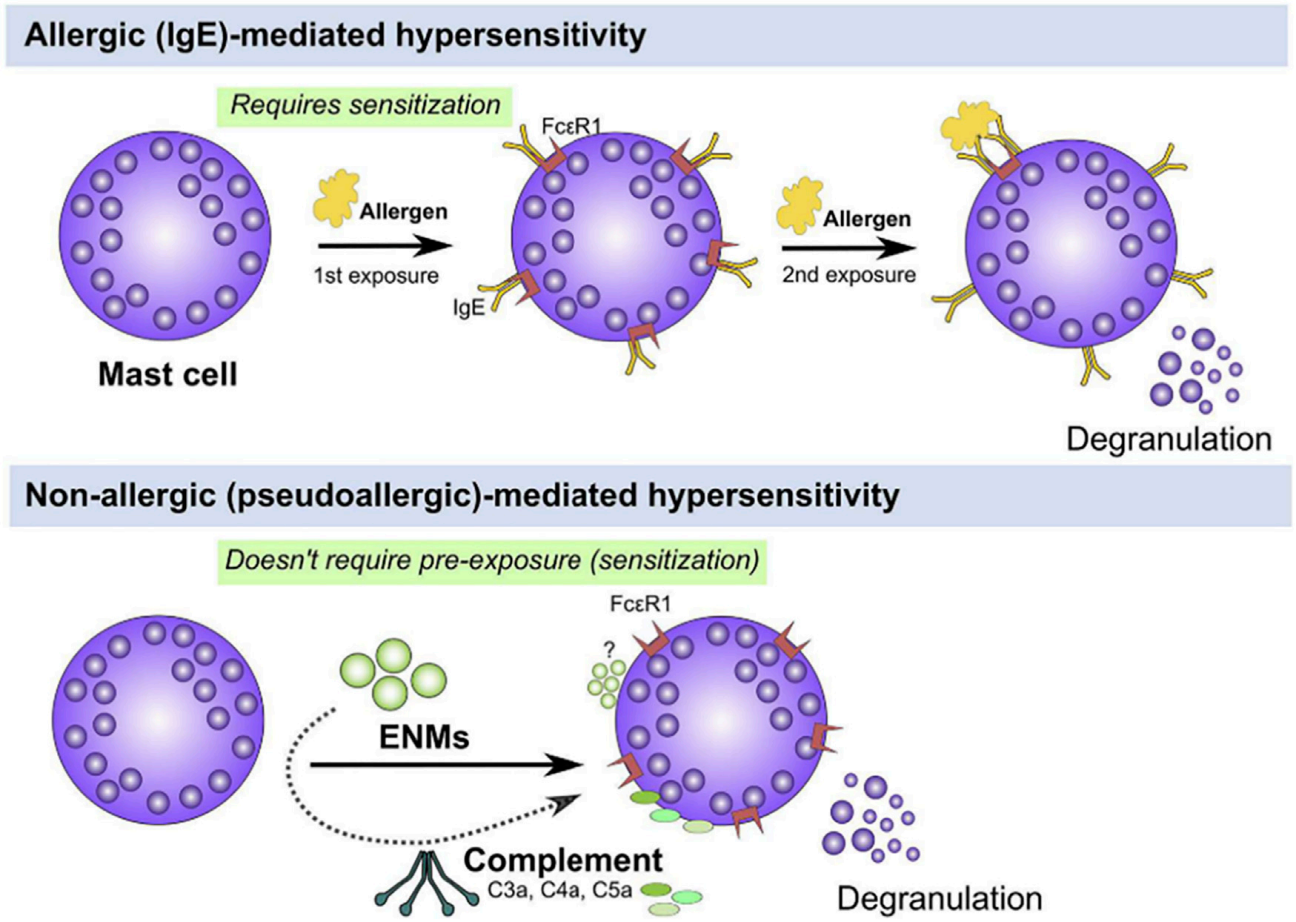
Hypersensitivity reactions: IgE-mediated and non-IgE-mediated. Mast cells can be activated either through IgE or non-IgE pathways. IgE stimulation (top panel) requires pre-exposure to an antigen (*e.g.*, pollen, mite, etc.) and upon a second exposure, IgE-bound FcεR1 are crosslinked leading to activation of signal transduction pathways that culminate in cell degranulation and activation (top panel). Mast cells can be also activated through non-IgE pathways (bottom panel) by a wide range of materials (*e.g.*, cytokines, complement fragments, basic polypeptides, environmental toxicants, toxic venoms, etc) which do not require prior exposure and may lead to mast cell degranulation and/or activation (bottom panel). Figure and captions are taken from ref (Alsaleh and Brown, 2020).

For a long time, PEG has been considered non-immunogenic (Richter and Åkerblom, 1983; Richter and Åkerblom, 1984). However, hypersensitivity reactions have been reported in patients receiving PEGylated therapies (Wenande and Garvey, 2016). These reactions have been classified as IgE-mediated (type I reaction) anaphylactic reactions and thus require a prior exposure (sensitization) to an allergen (antigen). After this first contact, mature B cells (plasma cells) produce IgE antibodies, which are bound to the receptors expressed on mast cells. Upon the second exposure, binding of the antigen to the surface-bound IgE receptor triggers the crosslinking of these receptors with degranulation and activation of mast cells, and the release of mediators of allergy (Alsaleh and Brown, 2020; Murphy et al., 2016) (Figure 5). In the case of PEG, this type of hypersensitivity reaction has been observed even in a healthy population that has not been treated with PEGylated drugs before (Mohamed et al., 2019; Hsieh et al., 2018; Garay et al., 2012) where the presence of pre-existing anti-PEG antibodies has been ascribed to the use of PEG as additives in dairy products (Mohamed et al., 2019; Zhou et al., 2021). Following the approval of Doxil by FDA in 1995, some hypersensitivity reactions were identified in patients treated with these PEGylated formulations that did were not explained by previously described mechanisms. Blood analysis collected soon after the administration of Doxil showed fragments from the complement cascade and the complement activation was proposed as a mechanism behind these hypersensitivity reactions (Chanan-Khan et al., 2003; Szebeni et al., 2011). These non-IgE-mediated hypersensitivity reactions are known as complement activation-related pseudoallergy (CARPA), with symptoms very similar to the type I hypersensitivity reaction, though they occur upon first contact without prior exposure, and lack a specific allergen, which is why it is called “pseudoallergy” (Szebeni, 2014; Szebeni, 2005; Szebeni, 2016) (Figure 5). This type of reaction was first described in the 1970s, caused by the radiocontrast agents (Lang et al., 1976; Kolb et al., 1978), but it was not until 2001 that it was recognized as a new type of hypersensitivity reaction (Szebeni, 2005). CARPA has been proposed as a possible cause of the anaphylactic reactions reported upon administration of the Pfizer/BioNTech and Moderna COVID-19 vaccines (Richter and Åkerblom, 1983; CDC, 2020; CDC, 2021; Jeimy et al., 2021; Klimek et al., 2021; Lim et al., 2021) although the reported cases are extremely few (Alhumaid et al., 2021). PEG has been postulated as a potential allergen causing these reactions (Risma et al., 2021; Zhou et al., 2021). Nevertheless, while PEG IgE antibodies have not been found in samples of patients with these reactions (Lim et al., 2021; Warren et al., 2021), the mechanism giving rise to CARPA in vaccinated individuals is not fully understood and it remains unclear whether PEG triggers these reactions (Bigini et al., 2021).

### Complement Activation-Related Pseudoallergy

**FIGURE 6:**
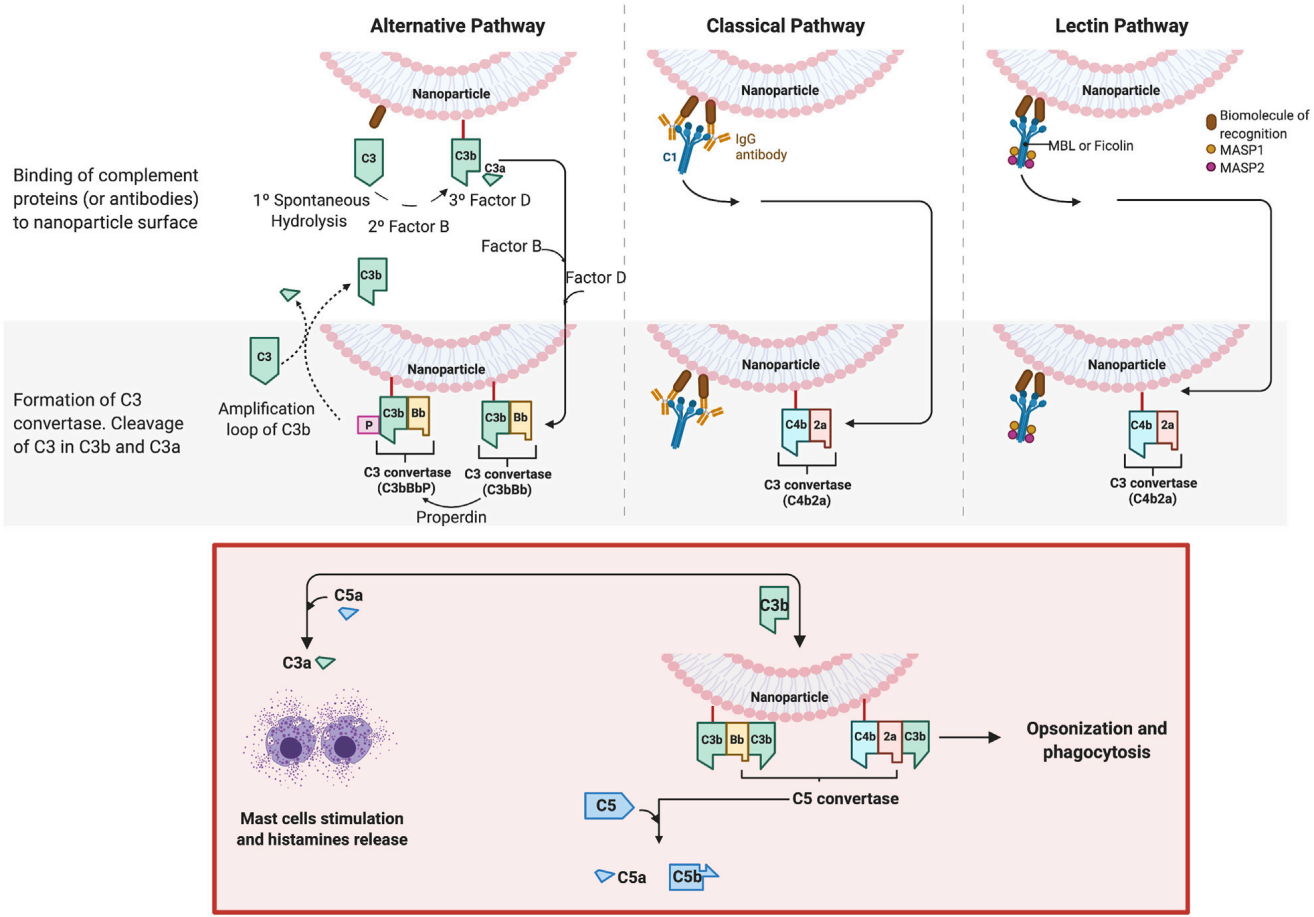
Complement activation by nanoparticles. The activation of the complement cascade occurs *via* three different mechanisms: the classical, alternative and lectin pathways. The classical and lectin pathway is initiated by the recognition of patterns on the particle surface by soluble mediators in the blood. For the classical pathway, the mediators are antibodies, IgG or IgM. The Fc portion of these antibodies is recognized by the complement component C1, which includes C1q (recognition protein) associated with the proteases C1r and C1s. The binding leads the production of C3 convertase (C4b2a) (central panel). For the lectin pathway, the C3 convertase also is produced, but in this case, the recognition is carried out through other mediators, lectins, which recognizes sugars (mainly N-acetyl glucosamine and mannose). In humans, five different lectins that can initiate this pathway have been identified: MBL, M-, L-, and H-ficolins, and collectin 11 (CL11 or CL-K1), which are associated with other proteins, such as MASP-2 and MASP-1 (right panel). The synthetized C3 convertase cleaves the abundant serum protein C3 into C3a and C3b. Later, a larger fragment, C3b, is covalently attached to the surface. On the other hand, when the spontaneous hydrolysis of the thioester bond in the protein C3 takes place, the alternative pathway (left panel) is initiated. C3(H20) is thus produced, which binds to the factor B, another plasma protein, to form C3(H20)B. The latter is cleavage by the factor D, producing the convertase C3(H20)Bb, also called fluid-phase C3 convertase, which will cleave C3 in C3a and C3b. In turn, this forms C3b, which binds to factor B and then, in turn, is cleaved by factor D, forming C3bBb. C3bBb is stabilized by another complement protein, known as properdin (P), and forms the convertase C3bBbP, able to cleave C3 into C3a and C3b, establishing the amplification of more C3b that could bind on the particle (or pathogen) surface. C3(H2O) also can deposit (non-covalently) on surfaces and initiate C3 cleavage (Murphy et al., 2016; Moghimi and Simberg, 2017). The figure was prepared in BioRender.

**FIGURE 7:**
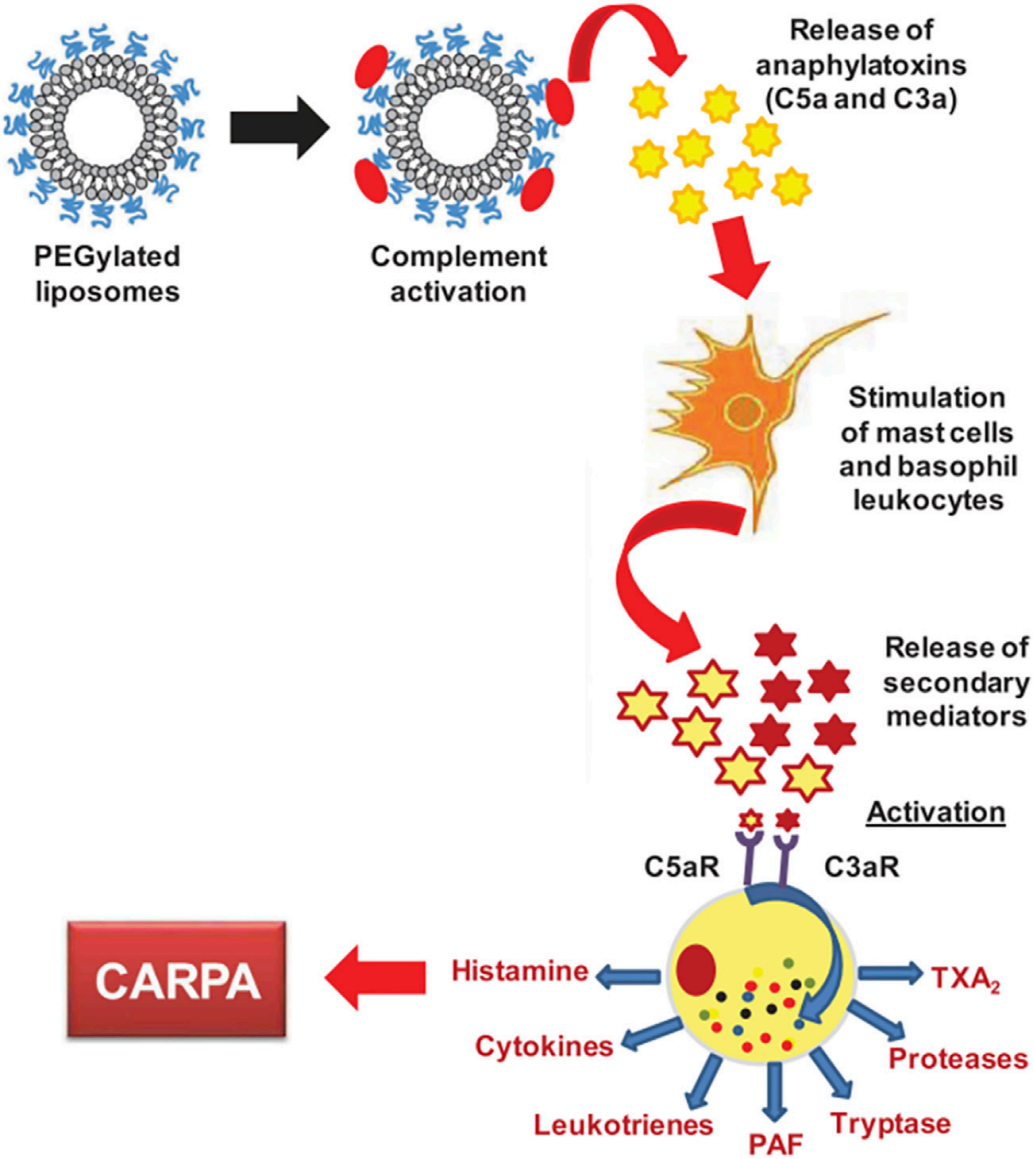
CARPA mechanism. The figure is taken from ref (Mohamed et al., 2019).

As described in a previous section, NPs in contact with a biological medium, such as the blood, interact with the protein, leading to the formation of a bio-corona. One important family of such proteins are the complement proteins, and these proteins have been identified on the corona of several classes of NPs, such as iron oxide (Vu et al., 2019), silica (Park et al., 2020), lipid-polymer hybrid (Salvador-Morales et al., 2009), polymeric and, of particular relevance, PEGylated liposomes (Chanan-Khan et al., 2003; Szebeni et al., 2012). Complement proteins represent 5% of the blood proteins and the complement family is composed of more than 30 different proteins. Overall, these proteins “complement” antibodies in terms of promoting phagocytic clearance of foreign entities (pathogens). The binding of these proteins on the NPs may triggers the complement activation cascade by three different mechanisms (classical, alternative, lectin) (Figure 6), depending on the physicochemical properties of NPs (size, morphology, surface patterns) and on the PEG composition and conformation (Ilinskaya et al., 2019; Hamad et al., 2010; Pannuzzo et al., 2020). The classical and lectin pathway is initiated by the recognition of patterns on the NP surface by soluble mediators in the blood, such as antibodies, IgG or IgM, and sugars (mainly N-acetyl glucosamine and mannose), respectively. On the other hand, when the spontaneous hydrolysis of the thioester bond in the protein C3 takes place, the alternative pathway is initiated. These mechanisms have been well described by Moghimi et al. (Moghimi and Simberg, 2017; Simberg and Moghimi, 2020). The complex proteolytic cascade generates the release of complement proteins such as iC3b, which enhance macrophage recognition, and the anaphylatoxins C3a, C4a and C5a (Moghimi and Szebeni, 2003). CARPA is triggered when the released anaphylatoxins interact with receptors in granulocytes, mast cells and monocytes, activating them and causing the release of vasoactive inflammatory mediators, involving tryptase, histamine, platelet-activating factor and other chemical molecules. These mediators interact with receptors in endothelial cells and muscle cells, triggering their activation and, finally, CARPA (Mohamed et al., 2019; Ilinskaya et al., 2019) (Figure 7). Even though PEG has been demonstrated to reduce protein adsorption, an incomplete surface coverage with the PEG polymer may still allow the attachment of biomolecules, and may promote the complement cascade by PEGylated NPs (Pelaz et al., 2015; Chen et al., 2017; Escamilla-Rivera et al., 2019; Pannuzzo et al., 2020). However, although the mechanism is not still clear, it has been demonstrated that the deposition of complement proteins on NP surfaces could be promoted by the presence of non-specific proteins in the protein corona, as opposed to binding of the complement proteins directly to the particle surface (Andersson et al., 2005; Chen et al., 2017). For instance, immunoglobulins such as IgG in the bio-corona of superparamagnetic iron oxide “nano-worms” and liposomes could trigger complement activation *via* the alternative pathway (Vu et al., 2019). The bio-corona formed on PEGylated doxorubicin-encapsulated liposomes in human cancer patients was elucidated in a recent study (Hadjidemetriou et al., 2019). The authors could show that immunoglobulins, lipoproteins, and complement proteins were the most abundant classes of proteins, contributing to 28%, 9%, and 4% of the total protein content respectively. It may be worthwhile to study the potential differences in protein corona composition between different COVID-19 mRNA vaccines. However, more research is needed to decipher the role of individual proteins in the bio-corona.

### Accelerated Blood Clearance and Immunogenicity

**FIGURE 8:**
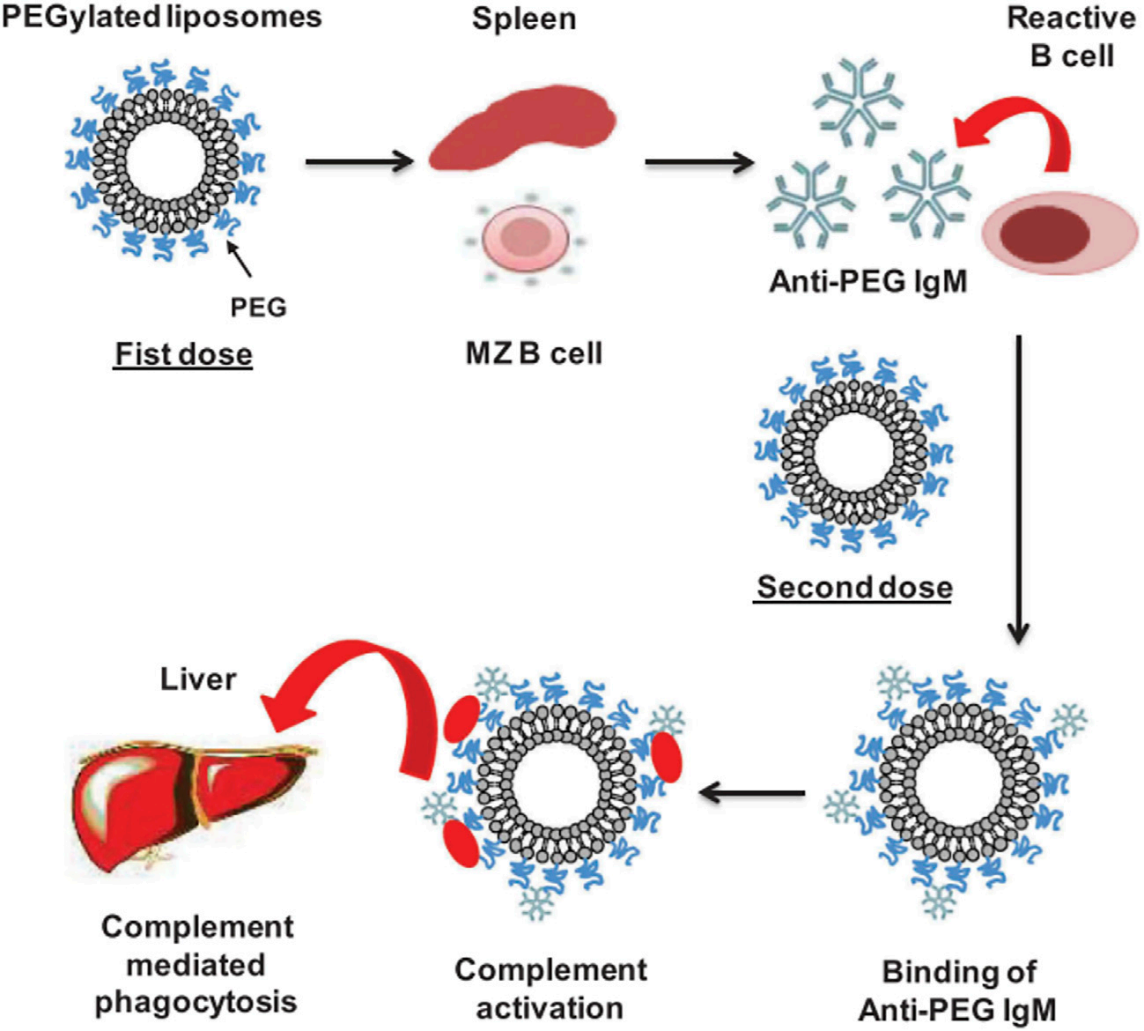
ABC mechanism. The figure is taken from ref (Mohamed et al., 2019).

In addition to the complement activation as a mechanism that may remove the NPs from the bloodstream, the ABC phenomenon, proposed by Dams et al., in 2000, explains how the production of anti-PEG IgM antibodies in the spleen, following the administration of an initial dose of PEGylated liposomes, influences and enhances the clearance of these NPs when subsequent doses are administered (Mohamed et al., 2019; Dams et al., 2000; Ishida et al., 2003; Ishida and Kiwada, 2008). The production of anti-PEG IgM antibodies has been demonstrated to be produced by B cells in the marginal zone of the spleen, it is independent of T cells and does not induce memory B cells (Ichihara et al., 2011; Semple et al., 2005). When the first dose of PEGylated liposomes is administrated, they reach the spleen and promote the production of anti-PEG IgM antibodies by crosslinking with the B cell receptor (BCR) and without the assistance of T cells. This leads to the existence of antibodies in the blood that, after a second injection (within the 5–21 days after the first dose), are bound to the PEGylated liposomes, promoting their uptake by macrophages (Figure 8). Several reports have also identified the presence of anti-PEG IgG antibodies (Chen et al., 2016; Yang et al., 2016; Kozma et al., 2019; Kozma et al., 2020). In this regard, PEG has been compared to a hapten, *i.e.*, a small molecule that elicits an immune response only when attached to a large carrier such as a protein (Murphy et al., 2016)**.** It is noted that the carrier alone may also be non-immunogenic. In general, molecules with an MW < 1,000 Da exhibit very weak immunogenicity. Such molecules coupled to a carrier protein will produce IgG antibodies with binding constants in the region of 10^–9^ M. Molecules with larger MW can produce IgG antibodies with much stronger binding constants in the range ∼10^–12^ to 10^–14^ M if they remain in circulation long enough in the blood (Landry et al., 2015). The produced antibodies are a combination of antibodies against different components of the PEGylated carrier and the immunogenicity and will depend on features of both PEG and carrier (Kozma et al., 2020). In this context, it is important to consider that PEGylated NPs may reduce protein adsorption to NPs, but this does not prevent protein adsorption altogether. Thus, PEG and (denatured) proteins will co-exist on the surface of the particles which may enhance immunogenicity. It is also notable that PEGylated surfaces have been reported to specifically adsorb IgM and IgG (Schellekens et al., 2013). This, therefore, makes it difficult to validate specific anti-PEG antibody binding.

Since PEGylated liposomes have been reported to trigger the production of antibodies by B cells without the assistance of T-helper cells (Ichihara et al., 2011; Hou et al., 2021), such NPs could potentially act as two different types of antigens, T-independent-1 (TI-1) and T-independent-2 (TI-2) antigens (Wang et al., 2007; Ichihara et al., 2011; Szebeni, 2016). TI-1 antigens activate B cells in a concentration-dependent manner: at high concentration, the signal is sufficient to induce proliferation and antibody secretion without specific antigen binding to immunoglobulin. At low concentration, only B cells specific for the antigen are able to bind and, therefore, this produces a specific antibody response to epitopes on the antigen (Murphy et al., 2016; Szebeni, 2016). On the other hand, TI-2 antigens are molecules with highly repetitive structures and activate mature B cells through the crosslinking of B cell receptors. It is also conceivable that specific metabolites of PEG could play a role in stimulating immune responses.

### Differences in Clinical Presentation of Anaphylaxis and CARPA

Both anaphylaxis and pseudoanaphylaxis present identical symptoms including breathing difficulties, urticaria, reduced blood pressure and loss of consciousness, and the clinical management of these patients is also similar in the acute phase. However, anaphylaxis is IgE-mediated and occurs rapidly after exposure to the relevant antigen. Recovery is also usually rapid (within 24 h). CARPA, on the other hand, is IgG and IgM activated and the initial onset tends to be slower. Treatment may be required over 4 weeks to ensure complete recovery. Examples of relevant immunological biomarkers for CARPA evidenced post-vaccination have been reported in Lim et al. (Lim et al., 2021). The results reflect the observations presented in this review. A simple IgE blood test readily distinguishes anaphylaxis from CARPA or pseudoanaphylaxis.

### Location, Location, Location: Intramuscular Complement Activation?

The discussion above refers essentially to complement activation in the blood. However, mRNA vaccines are administered through intramuscular (i.m.) injections. This raises the question as to whether the vaccines or their constituents reach the blood and if not, whether there is any likelihood of a complement-mediated adverse reaction being initiated in skeletal muscles. First, many vaccines are administered i. m. and trigger immune responses mainly in the draining lymph nodes (Irvine et al., 2020). However, inadvertent intravenous injection of a vaccine is possible, and this has been documented in the literature. Thus, among 164 registered nurses, forty percent reported having aspirated blood at least once (Thomas et al., 2016). Interestingly, a recent animal study has shown that i. v. but not i. m. injection of the Pfizer/BioNTech mRNA vaccine can induce acute myopericarditis (Li et al., 2021b). Furthermore, a preprint publication provided evidence that i. v. injection of an adenoviral vaccine might lead to platelet-adenovirus aggregate formation leading to platelet clearance and thrombocytopenia (Nicolai et al., 2021). Moreover, through the infiltration of the axillary lymph nodes, the mRNA vaccines could reach the vena cava *via* the thoracic duct (Dézsi et al., 2022). Taken together, accidental i. v. administration of a vaccine intended for i. m. administration could potentially trigger adverse events. The activation of complement in circulation is thus plausible, though it remains to be shown in vaccinated patients displaying adverse effects.

Skeletal muscle is the most abundant tissue in the human body and the biosynthesis of complement has been demonstrated in skeletal muscle cells (Syriga and Mavroidis, 2013). Moreover, studies have shown that complement plays a role in skeletal muscle regeneration (Zhang et al., 2017). It is presently a matter of conjecture, but it remains possible that mRNA vaccines may activate the complement cascade *in situ* following i. m. administration and/or trigger complement activation in the blood.

## Beyond PEG: Other Factors Involved in Anaphylaxis

PEG as a possible cause of the complement activation and the observed anaphylactic reactions has been widely discussed. However, it is important to consider whether other factors could be involved. For instance, as pointed out by Moghimi (Moghimi, 2021) in a recent commentary, some components of the Pfizer and Moderna vaccines, such as cholesterol and the phospholipid DSCP (1,2-distearoyl-snglycero-3 phosphocholine) (Table 1), may trigger complement activation *via* classical pathway through the production of natural (or induced) antibodies. Szebeni et al. (Szebeni et al., 2000) reported that the presence of cholesterol in LNPs over a concentration of 45 mol% leads to an increase of the pulmonary arterial pressure as a result of complement activation, whereas formulations containing under 45 mol% had no influence on pulmonary reactions. It was proposed that the interaction between naturally occurring anti-cholesterol antibodies and microcrystal aggregates formed by excess cholesterol trigger complement activation thereby manifesting as increased pulmonary hypertension through the release of anaphylatoxins. Furthermore, other positively charged components or the mRNA have been also identified as possible factors could activate the complement (Dézsi et al., 2022). Notably, the latter study showed that incubation of the Pfizer/BioNTech mRNA vaccine with pig serum *ex vivo* resulted in significant elevation of C3a anaphylatoxin and sC5b-9, the terminal complement complex.

In another recent study, it was shown that LNPs, alone or complexed with control noncoding poly-cytosine mRNA, are highly inflammatory in mice (Ndeupen et al., 2021). The authors speculated that innate immune memory elicited by the LNPs might form after the first vaccination (in humans) and that this could explain the more robust inflammatory responses upon the second vaccination. However, it is noted that inflammation could also underlie the adjuvant effect of vaccines and that it is not *a priori* detrimental. LNPs contain ionizable lipids (Table 1), *i.e.*, lipids that are positively charged at acidic pH to condense the RNA into LNPs and neutral at physiological pH to minimize toxicity in comparison to permanently charged lipids (Rietwyk and Peer, 2017). Efforts are under way to develop biodegradable ionizable lipids to reduce any long-term effects (Han et al., 2021).

Complement activation alone (or low-level complement activation) may not explain all instances of observed anaphylactic reactions. Hence, in one study in which Doxil was administrated intravenously to cancer patients, complement activation was measured according to the plasma sC5b-9 levels. The authors found that moderate to severe hypersensitivity occurred in 45% of patients while plasma SC5b-9 at 10 min after infusion was significantly elevated in 92% of reactors versus 56% in the non-reactor group (Chanan-Khan et al., 2003). Thus, it was not possible to establish a direct correlation between released fragments in the complement cascade, such as sC5b-9, and the observed reactions. In this regard, anaphylaxis may be explained due to the interaction of immunoglobulins (IgG) with FcR receptors on the immune cells and the platelet-activating factor (PAF) pathway (Strait et al., 2002; Wibroe et al., 2017; Moghimi, 2018). Immunoglobulins recognizing phospholipids may also trigger anaphylactic reactions through the interaction with FcR receptor on these cells (Moghimi, 2018).

The study by Wibroe et al. (Irvine et al., 2020) is of particular interest as the authors could show, using a set of polystyrene particles of varying geometries, that clearance of nanoparticles by pulmonary intravascular macrophages (PIMs) plays a key role in mediating adverse cardiopulmonary distress in pigs. The study implies that complement assessment alone may not be a sufficiently sensitive approach to predict adverse injection reactions insofar as the adverse reactions may occur in a complement-independent manner. However, PIMs exist in pigs, but not in humans (Irvine et al., 2020).

## Safe-by-Design Approaches for Nanomedicines

General procedures for safe-by-design of both nanomedicines and nano-enabled implantable medical devices have been described recently in the frame of the Horizon 2020 project, BIORIMA (Giubilato et al., 2020). The procedures require the evaluation of all nanoscale materials from raw materials, intermediates and final products to be subjected to a range of nanotoxicological tests, *e.g.*, cytotoxicity, genotoxicity, and immunotoxicity tests. In addition to the latter, safe-by-design nanopharmaceuticals requires the development of at-line and online high-functionality characterization and measurement of critical nanomaterial components for ensuring consistency of safe nanointermediates and final nanoscale products. This is known as Process Analytical Control (PAC) and is enshrined in FDA and EMA guidelines from 2003 onwards. Based on our analysis of the FDA and EMA submissions, routine quality assurance and control measures were used in the vaccine manufacturing process, but there was no information presented to indicate whether the PAC procedures were applied. Moreover, lessons learned in the nanosafety field should be considered including the role(s) of bio-corona formation on nanoparticles (Docter et al., 2015). It is also important to further investigate the use of PEG, and possible alternatives to PEG. Studies of biomimetic approaches including cell membrane “cloaking” may also prove very useful. Other critical aspects of the evaluation of nanomedicines including the importance of sterility are discussed elsewhere (Hannon and Prina ‐ Mello, 2021). Finally, given that the vaccination protocols now require repeated booster vaccinations, studies should be initiated to assess potential long-term effects of repeated exposures to the same or similar mRNA vaccines. For instance, whether a progressive build-up of anti-PEG IgG might negate the efficacy of the vaccines.

## Conclusion

The authorization and roll-out of the mRNA vaccines manufactured by Pfizer/BioNTech and Moderna has played a significant role in tackling the COVID-19 pandemic. The investigations carried out for decades using PEGylated liposomes as a drug delivery system, together with the evolution of mRNA technology, thus paved the way for the highly anticipated COVID-19 vaccines in a remarkably short time frame while retaining an excellent safety profile and also opens for new opportunities where nanotechnology can tackle other unmet clinical challenges. However, we have learned that after the application of the first dose, rare anaphylactic symptoms may occur, and this adverse effect of the vaccines has been tentatively attributed to the PEGylated LNPs, which may trigger complement activation and, possibly, CARPA. However, as there has been a higher incidence of adverse responses in patients suffering from existing allergies to drugs, they are highly monitored during the vaccine administration offering an excellent risk mitigation strategy. Complement activation by nanomedicines, including PEGylated liposomes, has been previously described and is believed to be associated with protein adsorption on the surface of the nanoparticles. Although the mechanism of these rare adverse reactions is still unknown, complement proteins as well as the presence of anti-PEG antibodies are believed to play an important role. These biomolecules are interconnected in what has been identified as the immune stimulatory vicious cycle (Szebeni, 2016), which produces adverse immunological reactions including CARPA. The anaphylactic reactions associated with COVID-19 vaccines are extremely rare and it is still unclear whether PEG molecules are the main culprit. More studies are needed to further improve on the safety profile of these vaccines as well as other nanomedicines.
