# Supplementary material for: Understanding the Role and Impact of Poly (Ethylene Glycol) (PEG) on Nanoparticle Formulation: Implications for COVID-19 Vaccines
Source: Front Bioeng Biotechnol. 2022 Jun 7;10:882363. doi: 10.3389/fbioe.2022.882363 (PMC9209764; doi:10.3389/fbioe.2022.882363)
Supplement: Supplementary file 1 [file Table1.docx]

Supplementary Material

**Supplementary Table 1. Use of PEGylated drugs/NPs approved by FDA and EMA.** (<https://www.fda.gov/> https://www.ema.europa.eu/en)

| M**edicine name** | **Approved by** **(year)** | **Therapeutic area** | **Active substance** | **MW PEG** **(kDa)** | **Pharmaco-therapeutic group** | **Administration** | **Marketing authorisation holder** |
| --- | --- | --- | --- | --- | --- | --- | --- |
| Skytrofa | FDA  (2021) | Growth hormone deficiency | Ionapegsomatropin-tcgd | 10 (x4) | Growth hormone receptor agonist | Subcutaneus | Ascendis |
| Empaveli | FDA  (2021) | Paroxysmal nocturnal hemoglobinuria (PNH) | Pegcetacoplan | 40 | Complement inhibitor | Subcutaneous infusion | Apellis |
| Nyvepria | EMA/FDA (2020/2020) | Neutropenia | Pegfilgrastim | 20 | Immunostimulants | Subcutaneus | Pfizer Europe MA EEIG |
| Cegfila | EMA  (2019) | Neutropenia | Pegfilgrastim | 20 | Immunostimulants | Subcutaneus | Mundipharma Corporation (Ireland) Limited |
| Grasustek | EMA  (2019) | Neutropenia | Pegfilgrastim | 20 | Immunostimulants | Subcutaneus | Juta Pharma GmbH |
| Besremi | EMA  (2019) | Polycythemia Vera | Ropeginterferon alfa-2b | 40 | Immunostimulants | Subcutaneus | AOP Orphan Pharmaceuticals AG |
| Esperoct | EMA/FDA (2019/2019) | Prophylaxis of bleeding in haemophilia A | Turoctocog alfa pegol | 40 | Antihemorrhagics | Intravenous | Novo Nordisk A/S |
| Palynziq | EMA/FDA (2019/2018) | Phenylketonurias | Pegvaliase | ~ 20 (x9) | Other alimentary tract and metabolism products | Subcutaneus | BioMarin International Limited |
| Ziextenzo | EMA/FDA (2018/2019) | Neutropenia | Pegfilgrastim | 20 | Immunostimulants | Subcutaneus | Sandoz GmbH |
| Fulphila | EMA/FDA (2018/2018) | Neutropenia | Pegfilgrastim | 20 | Immunostimulants | Subcutaneus | Mylan S.A.S |
| Pelgraz | EMA  (2018) | Neutropenia | Pegfilgrastim | 20 | Immunostimulants | Subcutaneus | Accord Healthcare S.L.U. |
| Pelmeg | EMA  (2018) | Neutropenia | Pegfilgrastim | 20 | Immunostimulants | Subcutaneus | Mundipharma Corporation (Ireland) Limited |
| Adynovi | EMA  (2018) | Prophylaxis of bleeding in haemophilia A | Rurioctocog alfa pegol | 20 | Antihemorrhagics | Intravenous | Baxalta Innovations GmbH |
| Udenyca | FDA  (2018) * | Neutropenia | Pegfilgrastim | 20 | Immunostimulants | Subcutaneus | ERA Consulting GmbH |
| Revcovi | FDA  (2018) | adenosine deaminase severe combined immune deficiency (ADA-SCID) | Elapegademase | ~ 5.6 (x13) | Immunostimulants | Intramuscular | Leadiant  Bioscience |
| Asparlas | FDA  (2018) | Acute lymphoblastic leukemia | Calaspargase pegol | 5 (x 31-39) | Antineoplastic agents | Intravenous | Servier Pharma |
| Jivi | EMA/FDA (2018/2017) | Prophylaxis of bleeding in haemophilia A | Damoctocog alfa pegol | 2x30 (x2) | Antihemorrhagics | Intravenously | Bayer AG |
| Refixia | EMA  (2017) | Prophylaxis of bleeding in haemophilia B | Nonacog beta pegol | 40 | Antihemorrhagics | Intravenous | Novo Nordisk A/S |
| Rebinyn | FDA  (2017) | Prophylaxis of bleeding in haemophilia B | Nonacog beta pegol | 40 | Antihemorrhagics | Intravenous | Novo Nordisk |
| Oncaspar | EMA/FDA (2016/1994) | Acute lymphoblastic leukemia  (component of antineoplastic combination therapy) | Pegaspargase | 5 (x 69-82) | Antineoplastic agents | Intramuscularly or intravenously | Les Laboratoires Servier |
| Adynovate | FDA  (2015) | Prophylaxis of bleeding in haemophilia A | Analogue of human plasma-derived Factor VIII | 20 (x ≥1) | Antihemorrhagics | Intravenous | Baxalta |
| Plegridy | EMA/FDA (2014/2014) | Multiple Sclerosis | Peginterferon beta-1a | 20 | Immunostimulants | Subcutaneus | Biogen Netherlands B.V. |
| Moventig, Movantik | EMA/FDA (2014/2014) | Opioid-induced constipation | Naloxegol | ~ 370 Da | Drugs for constipation | Oral  administration | Kyowa Kirin Holdings B.V./AstraZeneca |
| Lonquex | EMA  (2013) | Neutropenia | Lipegfilgrastim | 20 | Immunostimulants | Subcutaneus | Teva B.V. |
| Omontys | FDA  (2012) | Anemia due to chronic kidney disease | Peginesatide | 40 | Antianemic | Intravenous or subcutaneous | Takeda |
| Sylatron | FDA  (2011) | Melanoma | Peginterferon-alfa-2b | 12 | Immunostimulants | Subcutaneus | Merk |
| Asclera | FDA  (2010) | Varicose veins | Polidocanol | 600 Da | Vasoprotectives | Intravenous | Chemische Fabrik Kreussler |
| Krystexxa | FDA  (2010)* | Gout | Pegloticase | 10 (x 36) | Antigout preparations | Intravenous | Crealta Pharmaceuticals Ireland Limited |
| Cimzia | EMA/FDA (2009/2008) | Rheumatoid and psoriatic arthritis (in combination with methotrexate); axial spondyloarthritis; plaque psoriasis | Certolizumab pegol | 40 | Immunosuppressants | Subcutaneous | UCB Pharma SA |
| Mircera | EMA/FDA (2007/2007) | Anaemia associated with chronic kidney disease | Methoxy polyethylene glycol-epoetin beta | 30 | Antianemic preparations | Intravenous or subcutaneous | Roche |
| Macugen | FDA  (2004)* | Anti-angiogenic medicine in neovascular age-related macular degeneration | Pegaptanib | 20 (x2) | Ophthalmologicals | Intravitreous injection | PharmaSwiss Ceska Republika s.r.o |
| Somavert | EMA/FDA (2002/2003) | Acromegaly | Pegvisomant | 5 (x 4-6) | Pituitary and hypothalamic hormones and analogues | Subcutaneus | Pfizer Europe MA EEIG |
| Neulasta | EMA/FDA (2002/2002) | Neutropenia; Cancer | Pegfilgrastim | 20 | Immunostimulants | Subcutaneus | Amgen Europe B.V. |
| Pegasys | EMA/FDA (2002/2002) | Hepatitis C (in combination with other medicinal products), Hepatitis B | Peginterferon alfa-2a | 40 | Immunostimulants | Subcutaneus | Roche |
| PegIntron | FDA  (2001)* | Hepatitis C | Peginterferon alfa-2b | 12 | Immunostimulants | Subcutaneus | Merck Sharp & Dohme B.V. |
| Adagen | FDA  (1990) | ADA-SCID | pegademase bovine | 5 (x 11-17) | Immunostimulants | Intramuscular | Enxon |
| Onivyde pegylated liposomal | EMA  (2016) | Metastatic adenocarcinoma of the pancreas (in combination with 5-fluorouracil and leucovorin) | Irinotecan anhydrous free-base liposome | 2 | Antineoplastic agents | Intravenous | Les Laboratoires Servier |
| Caelyx pegylated liposomal | EMA  (1996) | Metastatic breast cancer; ovarian cancer; multiple myeloma (in combination with bortezomib); AIDS-related Kaposi’s sarcoma | Doxorubicin hydrochloride liposome | 2 | Antineoplastic agents | Intravenously | Baxter Holding B.V. |
| Doxil | FDA  1995) | Ovarian cancer; AIDS-related Kaposi’s Sarcoma; Multiple Myeloma (in combination with bortezomib) | Doxorubicin hydrochloride liposome | 2 | Antineoplastic agents | Intravenous | Schering |

*(drawback was reported for EMA)
